# Supplementary material for: Disruption of Sex-Linked Sox3 Causes ZW Female-to-Male Sex Reversal in the Japanese Frog Glandirana rugosa
Source: Biomolecules. 2024 Dec 9;14(12):1566. doi: 10.3390/biom14121566 (PMC11673724; doi:10.3390/biom14121566)
Supplement: Supplementary file 1 [file biomolecules-14-01566-s001.zip › Supplemental figures (tiff).pdf]

**Supplemental information (figures) for：**

# Disruption of Sex-Linked *Sox3* Causes ZW Female-to-Male Sex Reversal in the Japanese Frog *Glandirana rugosa*

Ikuo Miura, Yoshinori Hasegawa, Michihiko Ito, Tariq Ezaz and Mitsuaki Ogata

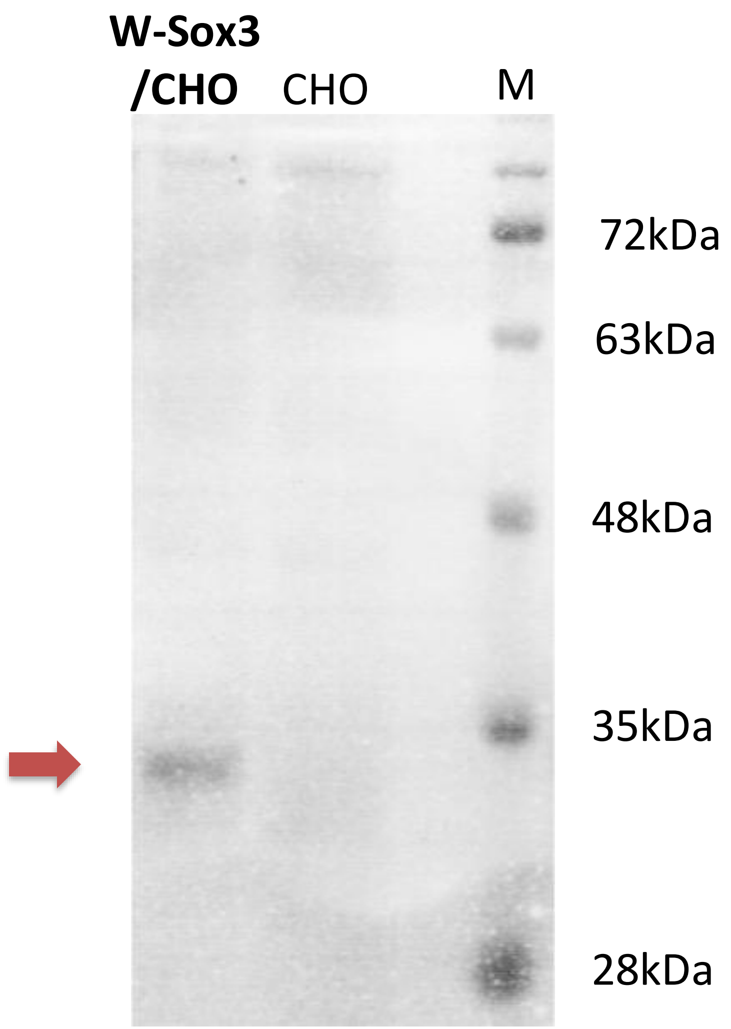


**Figure S1.** Western blotting of the proteins extracted from Chinese hamster cells transfected with *W-Sox3 of G. rugosa* construct. Red arrow indicates the band stained with anti-frog Sox3 antibody. Estimated molecular weight of Sox3 of *G. rugosa* is 30.8kD. M, molecular size marker

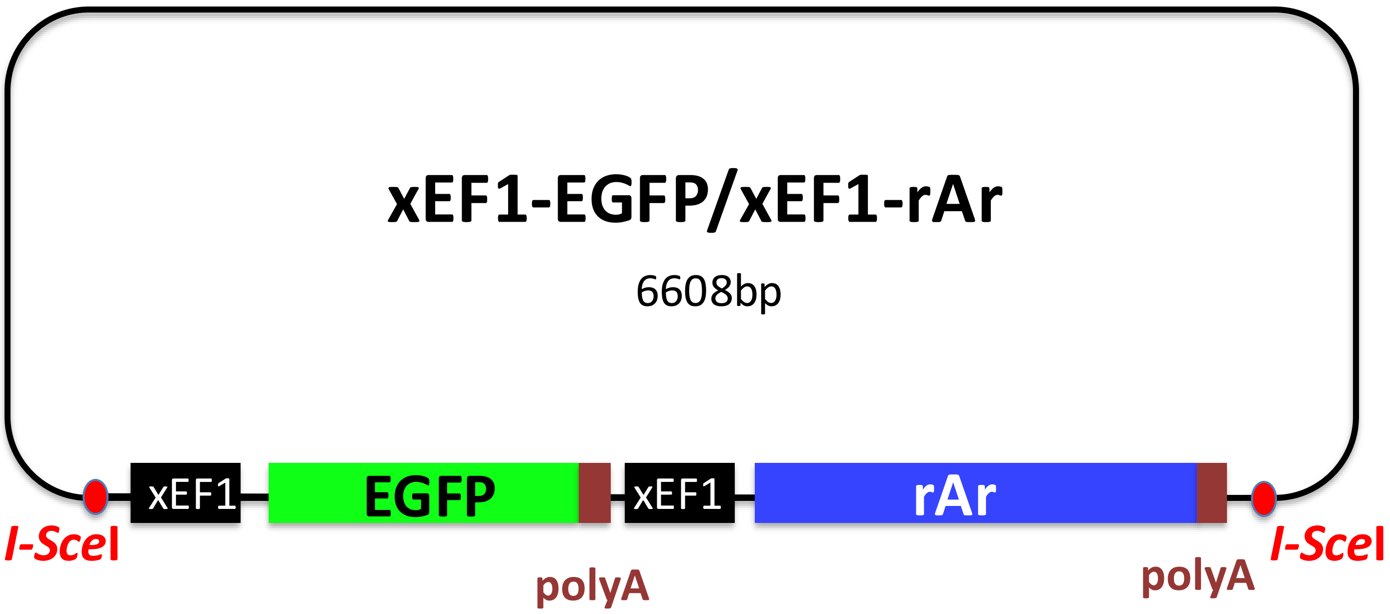


**Figure S2.** The transgenic construct including androgen receptor gene (*Ar*) under the promoter of *Xenopus laevis* elongation factor (*xEF*) and EGFP under the same promoter.

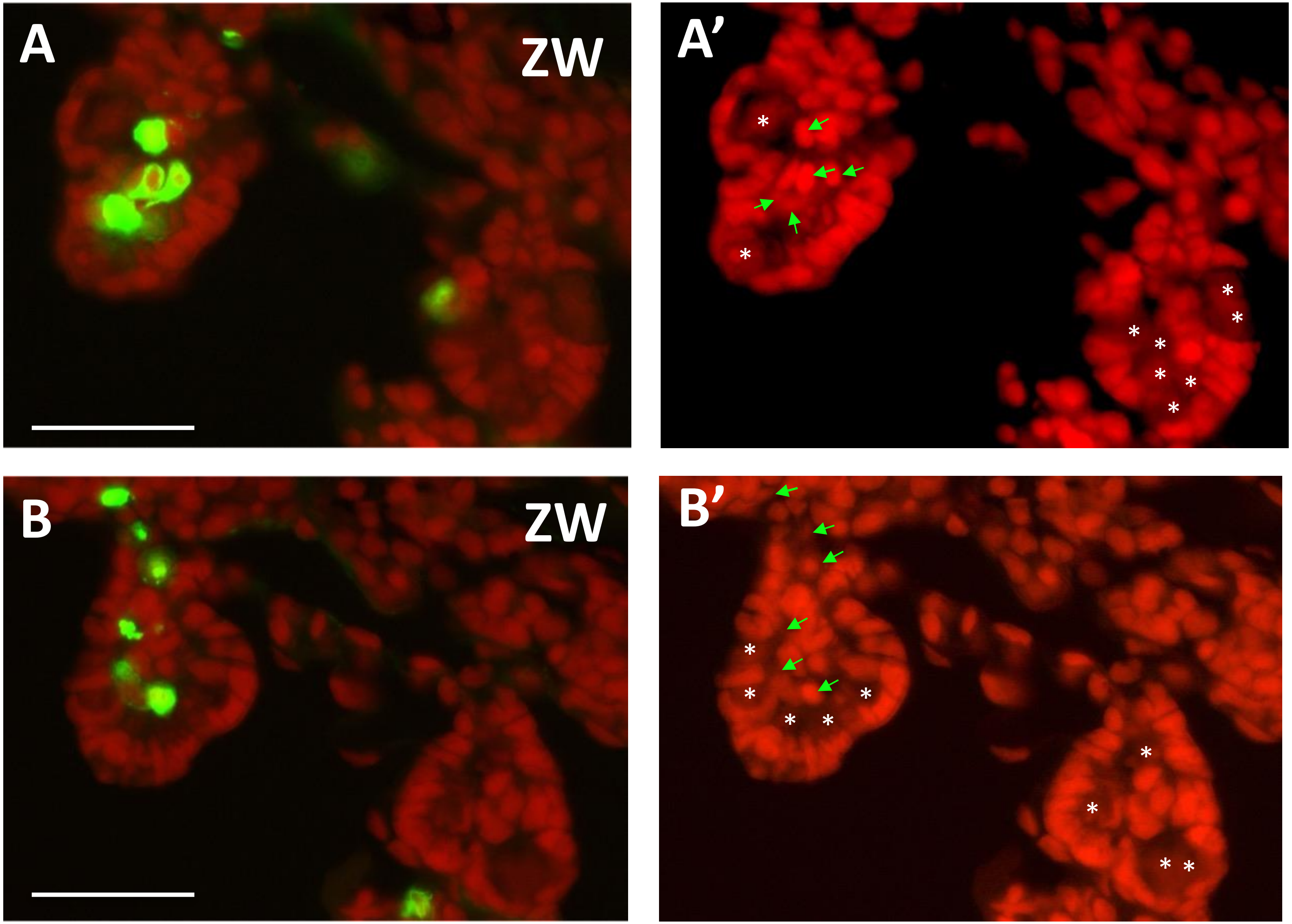


**Figure S3.** Localization of Sox3 protein in ZW tadpole gonads at 18 dpf. Merged pictures of gonads with Sox3 positive signals (A and B) and the gonads stained with PI before merging (A' and B'), of which nuclei are highlighted by increasing the contrast. Picture A is the same as picture D in Figure 4. White asterisks indicate germ cells with a larger cytoplasm, of which nuclei are faintly stained, and green arrows indicate the cells showing Sox3 protein signals.  The sequential positive signals in B suggest that the Sox3 expressing somatic cells migrate from the mesonephros into the ZW female gonad. Bar, 50 μm.

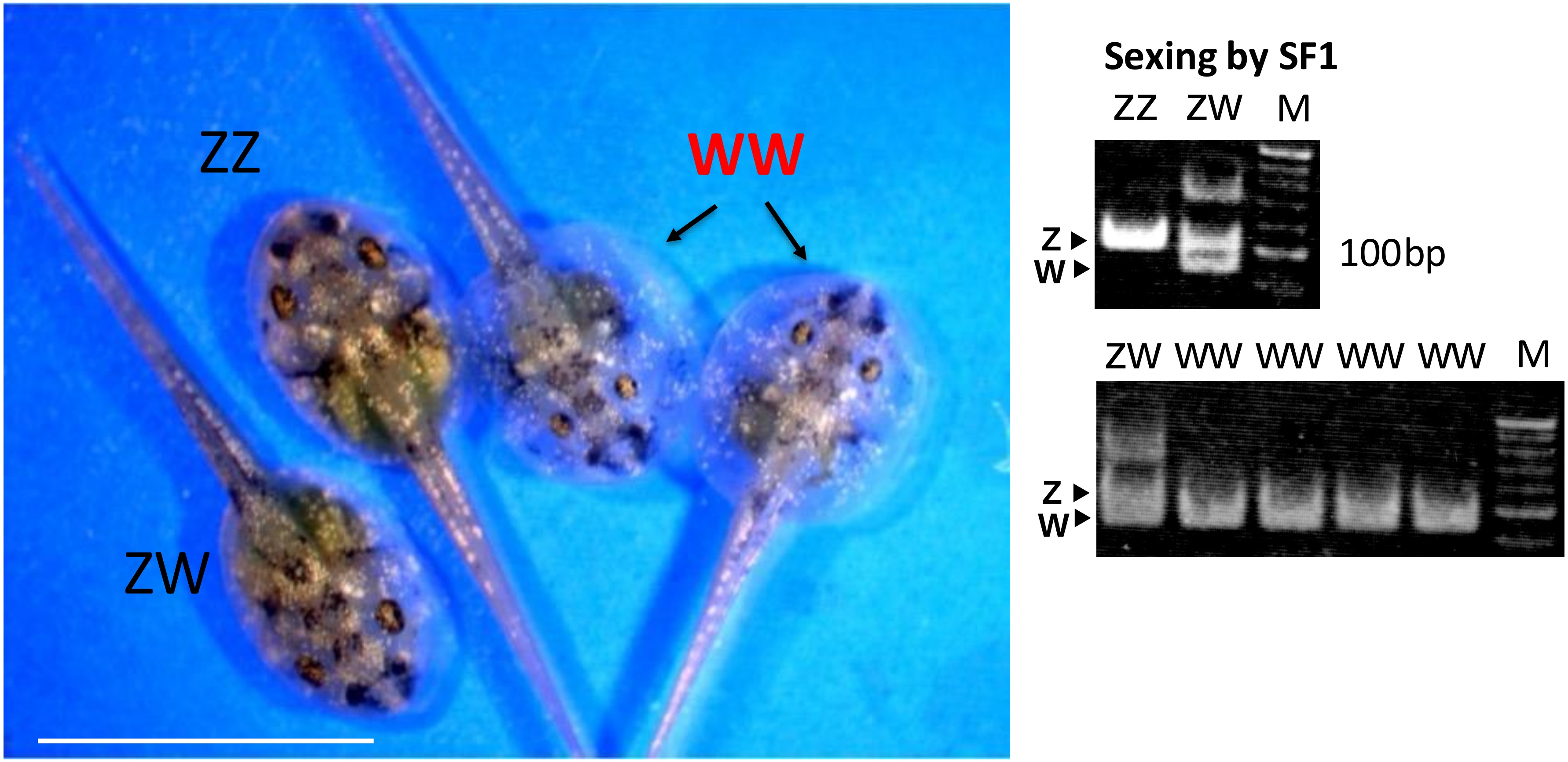


**Figure S4.** Tadpoles from the mating between ZW female and ZW sex-reversed male (3C-ZW1) and sexing. Red arrows on the left picture indicate WW tadpoles showing edema. Bar, 5mm. Right is the results of sexing using sex-linked *SF1*. ZZ, ZW and WW fragments are shown. M, molecular size marker.

# 1A-ZW1 hermaphrodite

## Right testis (28) 46.4%

```
                                  No. of
                                  clones
TGGACACTGATATCAAGAGCCC            15
TGGACACTGA------GAGCCC             1
TGGACACTGA--------GCCC             6
TGGACACTGAT----------C             1
                  T
                  v
TGGACACTGATATCAAGAGCCC             5
```

## Left ovary (23) 60.9%

```
                                  No. of
                                  clones
TGGACACTGATATCAAGAGCCC             9
TGGACACTGATG-CAAGAGCCC             1
TGGACACTGA--------GCCC             1
                  T
                  v
TGGACACTGATATCAAGAGCCC            10
                  TA
                  v
TGGACACTGATATCAAGAGCCC             2
```

**Figure S5–S10**. The mutations identified in or around the target sequences of *Sox3* in the ZW sex-reversed males and hermaphrodites. –, a nucleotide deletion. v, insertion position. a period, nucleotide sequence upstream or downstream the target sequence. The number of clones sequenced is shown in parenthesis. The target sequences are indicated in red and substituted nucleotides, in blue. The number of in-frame mutation is depicted in green.

**Figure S6**

# 1A-ZW2 hermaphrodite

## Right testis (22) 54.5%

| Insertion | Sequence | No. of clones |
|---|---|---|
| | TGGACACTGATATCAAGAGCC | 10 |
| | TGGACACTGATGTCAAGA-CC | 1 |
| | TGGACACTGATG-C-AGAGCC | 1 |
| | TGGACACTGATT--------CC | 1 |
| T | TGGACACTGATATCAAGAGCC | 4 |
| T | TGGACACTGATATCA-GAGCC | 1 |
| T | TGGACACTGAT-TCAAGAGCC | 1 |
| TCA | TGGACACTGATATCAAGAGCC | 1 |
| CGGGGCTCCCCAC; T | TGGACACTGA------GAGCC | 1 |
| CGGGGCTCCCC | TGGACACTGA---CA-GAGCC | 1 |

## Left ovary (14) 21.4%

| Insertion | Sequence | No. of clones |
|---|---|---|
| | TGGACACTGATATCAAGAGCCC | 10 |
| | TGGACACTGA------GAGCCC | 1 |
| T | TGGACACTGATATCAAGAGCCC | 2 |

**Figure S7**

# 1C-ZW1 hermaphrodite

| Right testis (16) 88.9% | No. of clones |
| --- | --- |
| CCTACATGAATGCAGCTTCTACCTACAG | 2 |
| CCTACATGAATCAGTCTTCTACCTACAG | 2 |
| CCTACATGAAT----CTTCTACCTACAG | 1 |
| CCTACATGAAT-------CTACCTACAG | 3 |
| CCTACA---29bp deletion-.........ACCAGC | 1 |
| ATGTCCCCCTCTCCCCTCTCCCC(23bp)<br>v<br>CCTACATGAATGCAGCTTCTACCTACAG | 9 |

| cDNA(13) 93.3% | No. of clones |
| --- | --- |
| CCTACATGAATGCAGCTTCTACCTACAG | 1 |
| CCTACATGAATCAGTCTTCTACCTACAG | 1 |
| CCTACATGAATGC--CTTCTACCTACAG | 2 |
| CCTACATGAATG---CTTCTACCTACAG | 2 |
| CCTACATGAAT----CTTCTACCTACAG | 1 |
| CCTACATGAATG-----TCTACCTACAG | 1 |
| CCTACATGAAT-------CTACCTACAG | 2 |
| ATGTCCCCCTCTCCCCTCTCCCC(23bp)<br>v<br>CCTACATGAATGCAGCTTCTACCTACAG | 5 |
| GTGCA..536bp–CAGCTTCTACCTACAG<br>deletion<br>(88-622) | 2 |

**Figure S8**

# 1C-ZW1 hermaphrodite

## Right ovary (24) 79.2%

| Sequence | No. of clones |
|---|---|
| CCTACATGAATGCAGCTTCTACCTACAG | 4 |
| CCTACATGAATGC-GCTTCTACCTACAG | 9 |
| TTCT...27bp deletion----------ACCTACAG | 7 |
| GGCA v CCTACATGAATGCAGCTTCTACCTACAG | 4 |

## cDNA(13) 84.6%

| Sequence | No. of clones |
|---|---|
| CCTACATGAATGCAGCTTCTACCTACAG | 2 |
| CCTACATGAATGC-GCTTCTACCTACAG | 7 |
| TTCT..27 bp deletion----------ACCTACAG | 3 |
| GGCA v CCTACATGAATGCAGCTTCTACCTACAG | 1 |

## Left ovary (19) 68.4%

| Sequence | No. of clones |
|---|---|
| CCTACATGAATGCAGCTTCTACCTACAG | 6 |
| CCTACATGAATCAGTCTTCTACCTACAG | 1 |
| CCTACATGAATGCAGC-TCTACCTACAG | 1 |
| CCTACATGAAT-CACCTTCTACCTACAG | 2 |
| CCTACATGAATGC-G-TTCTACCTACAG | 2 |
| CCTACATGAATGC---TTCTACCTACAG | 1 |
| CCTACATGAA-------------TACAG | 1 |
| CCT------------TCTACCTACAG | 1 |
| T v CCTACATGAATGCAGCTTCTACCTACAG | 2 |
| CC v CCTACATGAAT-CAGCTTCTACCTACAG | 1 |
| ATGTCCCCCTCTCCCCTCTCC v CCTACATGAATGCAGCTTCTACCTACAG | 1 |

Figure S9

# 3C-ZW1 male

## Right testis (21) 47.6%

| Sequence | No. of clones |
| --- | --- |
| AGACCTACATGAATGCAGCTTCTACCTAC | 10 |
| AGACCTACATGAATGCAGCCTCTACCTAC | 1 |
| AGACCTACATGAATGCA-CTTCTACCTAC | 1 |
| AGACCTACATGAAC----CTTCTACCTAC | 2 |
| AGACCTACATGAAT-----TTCTACCTAC | 3 |
| AGACCTGC-----------TTCTACCTAC | 1 |
| AG-----------------CTTCTACCTAC | 1 |
| AGACCTAC-----------------CTAC | 1 |
| AGACCTACATGAATAGCTTTTCTACCTAC (AT inserted, marked v) | 1 |

## cDNA(27) 14.8%

| Sequence | No. of clones |
| --- | --- |
| AGACCTACATGAATGCAGCTTCTACCTAC | 23 |
| AGACCTACATGAATG---CTTCTACCTAC | 3 |
| AGACCTACATGAAT---GCTTCTACCTAC | 1 |

Figure S10

# 2AC-ZW1 male

| Right testis (16) 62.5% | No. of clones |
|---|---|
| CCTACATGAATGCAGCTTCTACCTACAGCATGT | 6 |
| CCTACATGAATGCA-CTTCTACCTACAGCATGT | 1 |
| CCTACATGAATGT--CTTCTACCTACAGCATGT | 1 |
| CCTACATGAATGC---TTCTACCTACAGCATGT | 2 |
| CCTACATGAA-------GCTACCTACAGCATGT | 1 |
| CCTACATGAAT-------CTACCTACAGCATGT | 1 |
| CCT---------------TCTACCTACAGCATGT | 1 |
| CCTACATGAAT-------------------GT | 1 |
| CCTACATGAATG-------------------T | 1 |
| --404bp deletion—-----CTACCTACAGCATGT (226-628) | 1 |

| cDNA(13) 61.5% | No. of clones |
|---|---|
| CCTACATGAATGCAGCTTCTACCTA | 5 |
| CCTACATGAATGT—-CTTCTACCTA | 2 |
| CCTACATGAAT----CTTCTACCTA | 1 |
| CCTACAT----------TCTACCTA | 1 |
| -404bp deletion--------CTACCTA (226-628) | 4 |
